# Renal Cell Carcinoma–Associated Diabetes Mellitus Due to Paraneoplastic Syndrome in Maintenance Hemodialysis: A Case Report

**DOI:** 10.1016/j.xkme.2022.100477

**Published:** 2022-04-29

**Authors:** Yusuke Yoshimura, Tatsuya Suwabe, Daisuke Ikuma, Yuki Oba, Masayuki Yamanouchi, Akinari Sekine, Hiroki Mizuno, Eiko Hasegawa, Junichi Hoshino, Kei Kono, Keiichi Kinowaki, Kenichi Ohashi, Naoki Sawa, Yoshifumi Ubara

**Affiliations:** 1 Nephrology Center, Toranomon Hospital Kajigaya, Kanagawa, Japan; 2 Okinaka Memorial Institute for Medical Research, Toranomon Hospital, Tokyo, Japan; 3 Department of Pathology, Toranomon Hospital, Tokyo, Japan; 4 Department of Human Pathology, Graduate School of Medical and Dental Sciences, Tokyo Medical and Dental University, Tokyo, Japan

**Keywords:** Acute-onset diabetes, insulin resistance, homeostasis model assessment of insulin resistance, paraneoplastic syndromes, renal cell carcinoma

## Abstract

A 59-year-old Japanese woman with a 22-year history of long-term hemodialysis was admitted to our hospital for further examination of hyperglycemia and anemia. Five months before hospitalization, her fasting plasma glucose value was 99 mg/dL and her glycated hemoglobin was 5.7%. On admission, her fasting plasma glucose value was 873 mg/dL, glycated hemoglobin was 16.2%, C-peptide reactivity was 22.3 ng/mL (reference range, 0.5-3.0), and homeostasis model assessment of insulin resistance (HOMA-IR) was 10.6 (reference range, <2.0); the high HOMA-IR indicated high insulin resistance. Intensive insulin therapy was started for hyperglycemia, which required more than 40 units/day. Computed tomography showed a hypervascular lesion 2.2 cm in diameter on the right kidney; therefore, right nephrectomy was performed. Complete resection was confirmed, and the lesion was diagnosed as a clear cell type of renal cell carcinoma (RCC). Immediately after nephrectomy, glycemic control normalized and administration of insulin was discontinued. Fourteen days after nephrectomy, the HOMA-IR decreased to 2.96. RCC that develops in patients receiving long-term hemodialysis has been reported to be dialysis-related RCC, but there have been no reports suggesting a relationship between dialysis-related RCC and diabetes. To our knowledge, this is the first report of RCC presenting with the paraneoplastic syndrome of acute-onset diabetes because of insulin resistance.

## Introduction

Renal cell carcinoma (RCC) has been reported to be closely related to paraneoplastic syndromes, which subside only after nephrectomy and reappear if RCC metastasizes outside the kidney. Abnormalities in glucose metabolism have also been reported to be common in patients with RCC. Several case reports described hyperglycemia that resolved after nephrectomy for RCC. These findings have led to research into possible factors that may be either secreted or stimulated by the tumor. Hormones such as glucagon have been isolated from RCC tumor extracts.^1^ The above-mentioned reports described RCC in patients without chronic kidney disease, but here, we report a case of acute-onset diabetes and RCC that appeared after 22 years of receiving long-term hemodialysis.

## Case Report

A 59-year-old Japanese woman was admitted to our hospital for further examination of hyperglycemia. She developed nephrotic syndrome at the age of 31 years, and hemodialysis was started at the age of 37 years. She had no family history of relevant diseases. She had never smoked, and she drank only a little alcohol socially. Five months before her hospitalization, her fasting plasma glucose value was 99 mg/dL. She had no history of diabetes up to this time.

On admission, the patient was 150 cm tall and weighed 55.9 kg. Her blood pressure was 140/78 mm Hg, pulse rate was 65/min, and temperature was 36.0 °C.

Blood test results on admission are listed in Table 1. The complete blood count showed hemoglobin at 8.8 g/dL, leucocytes at 6,200/μL, and thrombocytes at 14.6 × 10⁴/μL. The results of blood chemistry tests were sodium at 129 mEq/L, potassium at 5.6 mEq/L, chloride at 96 mEq/L, C-reactive protein at 0.4 mg/dL, fasting plasma glucose at 873 mg/dL, glycated hemoglobin at 16.2%, total cholesterol at 152 mg/dL, and triglycerides at 177 mg/dL. The results of subsequent endocrinological serum testing were adrenocorticotropin at 31.7 pg/mL (reference range, 7.2-63.3 pg/mL), cortisol at 17.5 μg/dL (reference range, 6-12 μg/dL), C-peptide reactivity at 22.3 ng/mL (reference range, 0.5-3.0 ng/mL), anti-glutamic acid decarboxylase antibodies < 1.3 (reference range, <1.3), negative islet-cell antibodies (reference value, negative), and homeostasis model assessment of insulin resistance (HOMA-IR) at 10.6 (reference range, <2.0); the HOMA-IR indicated high insulin resistance (Table 1).

**Table 1:** Patient Laboratory Values

Laboratory Measure	On Admission	14 Days After Surgery	Reference Range	Unit
Erythrocytes	2.77 × 10⁶	3.08 × 10⁶	3.8-5.0 × 10⁶	/μL
Hemoglobin	8.8	9.9	11.3-15.0	g/dL
Hematocrit	25.4	30.3	33.9-45.0	%
Leucocytes	6,200	6,200	3,200-7,900	/μL
Thrombocytes	14.6 × 10⁴	18.7 × 10⁴	15.5-35.0 × 10⁴	/μL
Total protein	7.7	7.8	6.9-8.4	g/dL
Albumin	3.4	3.2	4.1-5.1	g/dL
Urea nitrogen	56	37	8.0-21.0	mg/dL
Creatinine	10	7.8	0.6-1.0	mg/dL
Uric acid	9	6.6	3.0-5.0	mg/dL
Sodium	129	136	140-146	mEq/L
Potassium	5.6	4.2	4.0-4.8	mEq/L
Chloride	96	100	90-96	mEq/L
Calcium	8.4	9.6	8.0-8.6	mEq/L
Phosphorus	6.3	3.7	4.0-5.0	mg/dL
C-reactive protein	0.4	0.9	<0.3	mg/dL
Total cholesterol	152	177	142-248	mg/dL
Triglycerides	177	205	40-149	mg/dL
Fasting glucose	873	88	80-90	mg/dL
Growth hormone	0.12	0.22	<5.0	ng/mL
Insulinlike growth factor 1	112	162	37.0-266.0	ng/mL
Adrenocorticotropin	31.7	67.2	7.0-60.0	pg/mL
Cortisol	17.5	15.2	6.0-12.0	μg/dL
Renin activity	<2.4	<2.4	3.4-21.3	pg/mL
Aldosterone	13.7	6.7	3.0-15.0	ng/dL
C-peptide reactivity	22.3	18.87	0.5-3.0	ng/mL
HOMA-IR	10.6	2.96	<2.0	

Abbreviation: HOMA-IR, homeostasis model assessment of insulin resistance.

**Figure 1:**
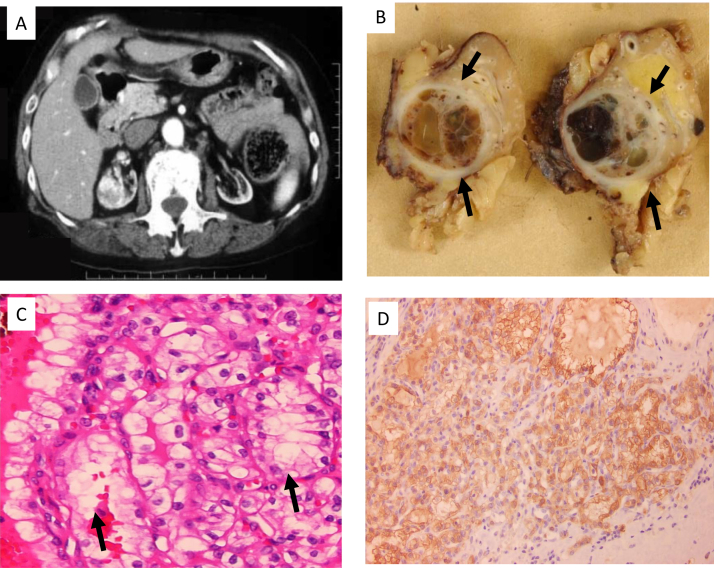
Imaging, histology, and immunohistochemistry findings. (A) A contrast-enhanced computed tomography scan showed a hypervascular lesion 2.2 cm in diameter on the right kidney. (B) The surgical specimen of the right kidney showed a mass lesion (arrows) with necrotic tissue inside. (C) Clear cell renal cell carcinoma (arrow) was diagnosed. (D) Immunohistochemistry staining of renal cell carcinoma was positive for neuron-specific enolase (brown-colored cells).

In a patient receiving long-term hemodialysis, the cause of rapidly increasing blood glucose was investigated. First, an ultrasound screening suggested a mass lesion in the left kidney. A contrast-enhanced computed tomography scan was added. There was a hypervascular lesion 2.2 cm in diameter on the right kidney (Fig 1A). Therefore, on day 25 after hospitalization, right nephrectomy was performed.

Complete resection was confirmed, and the lesion was diagnosed as clear cell type of RCC (Fig 1B). Immunohistochemistry staining was positive for neuron-specific enolase but negative for thyroid-stimulating hormone, corticotropin, glucagon, somatostatin, pancreatic polypeptide, chromogranin A, CD56, and CD57.

**Figure 2:**
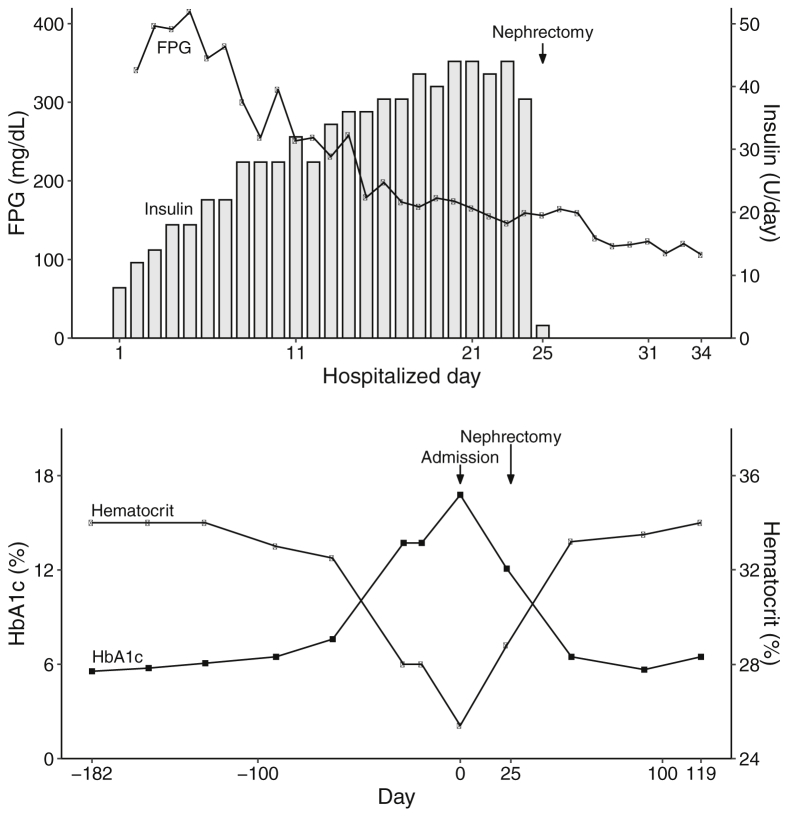
Clinical course. The upper field shows the relation between fasting plasma glucose (FPG) and insulin dose per day. The lower field shows the relation between glycated hemoglobin (HbA1c) (%) and hematocrit (%).

After hospitalization, intensive insulin therapy was started for hyperglycemia, which required more than 40 units/day. Immediately after nephrectomy, glycemic control normalized and administration of insulin was discontinued. After 14 days, the HOMA-IR decreased to 2.96 (Fig 2). At a follow-up after 9 years, the patient was still in a normoglycemic state and no longer required hypoglycemic therapy.

## Discussion

We described a case of acute-onset diabetes after long-term hemodialysis. The patient was diagnosed with clear cell type of RCC, and diabetes improved after nephrectomy. To examine the relationship between RCC and the development of diabetes, we searched for previous reports that dealt with these issues.

Spyridopoulos et al^2^ examined the association of RCC with diabetes and insulin resistance as assessed by HOMA-IR; RCC was positively correlated with the development of diabetes mellitus but negatively correlated with insulin resistance. Okada et al^3^ reported on an 83-year-old man with stable chronic kidney disease who exhibited a sudden increase in urinary protein excretion and poor glycemic control. The patient was diagnosed with RCC, and glycemic control improved after nephrectomy; immunohistochemistry confirmed that the RCC tissue was positive for interleukin-6 (IL-6). They suggested that the production of IL-6 in RCC may be related to the development of diabetes via insulin resistance, but the positivity of staining usually confirms only the overproduction of IL-6 and does not necessarily indicate elevated levels of serum IL-6.^3^ Blay et al^4^ reported that IL-6 associated with RCC was related to elevation of C-reactive protein, haptoglobin, thrombocytosis, neutrophilia, and monocytosis but did not mention abnormalities in blood glucose. Elias^5^ reported on a 52-year-old woman with new-onset diabetes. The tests for serum islet-cell antibody and anti-glutamic acid decarboxylase antibodies were positive, and the patient was diagnosed with left-sided RCC. After treatment by surgery, the patient’s blood glucose control improved and the autoantibody titer decreased. Elias^5^ suggested the significance of the serum anti-islet-cell antibody and anti-glutamic acid decarboxylase antibodies via RCC or the development of new-onset diabetes. Khanna et al^6^ reported on the occurrence of paraneoplastic hyperglycemia with a rare variant of RCC, the mucinous tubular and spindle-cell variant. The patient recovered well after surgery for RCC and maintained normal glycemic control without any hypoglycemic agents. Immunostaining for corticotropin, glucagon, insulin, and growth hormone was performed on the excised RCC tissue, and staining was positive for glucagon. Khanna et al^6^ hypothesized that the production of glucagon in RCC was related to the development of diabetes. Gapp et al^7^ reported on a 61-year-old man with new-onset hyperglycemia and papillary-type RCC. Hyperglycemia resolved after resection of RCC, so insulin therapy was discontinued; subsequently, the patient required only an oral hypoglycemic. Gapp et al^7^ could not prove any factors related to RCC and the development of diabetes. Based on previous reports, it is clear that RCC is associated with the development of diabetes. However, its pathogenesis varies widely, and it is difficult to find a common factor, but careful analysis of the cases may show the pathogenesis.

The onset of RCC is slow, but the onset of diabetes is rapid. There is no definitive answer to this explanation, but previous reports showed that the onset of paraneoplastic syndromes associated with RCC has been as rapid as in our case. It is well known that clinical symptoms occur only when the factors that induce diabetes exceed a certain threshold. Perhaps the symptoms appear only after the tumor has reached a certain size.^8^^,^^9^

Because some reports suggested a relationship between IL-6 and RCC,^3^^,^^10^ serum IL-6 levels were determined using stored sera at 3 points, 1 year before RCC diagnosis, at the time of RCC diagnosis, and 1 year after surgery, with measurements of 18.4 pg/mL, 189 pg/mL, and <7.0 pg/mL, respectively. There seemed to be a relationship between RCC and IL-6. However, the staining in RCC tissue was performed based on previous methods,^3^^,^^10^^,^^11^ but the staining of IL-6 for RCC was negative. It is unclear whether IL-6 has a close relation with RCC, which leads to paraneoplastic syndrome.

In conclusion, we experienced a case of acute-onset diabetes and RCC that became apparent after 22 years of long-term hemodialysis. HOMA-IR, fasting plasma glucose, and glycated hemoglobin values improved immediately after surgical nephrectomy for RCC. This case indicates that insulin resistance associated with the development of RCC can be attributed to the acute onset of diabetes. RCC that develops in patients treated with long-term hemodialysis has been reported as dialysis-related RCC, but there have been no reports suggesting a relationship between dialysis-related RCC and diabetes.^10^ To our knowledge, this is the first report of RCC in a patient treated with long-term hemodialysis with paraneoplastic syndrome characterized by acute-onset diabetes because of insulin resistance.
